# The active metabolites of *Eucommia ulmoides* leaves alleviate atherosclerosis induced by a high-fat diet and VD3 in rats

**DOI:** 10.3389/fphar.2025.1625200

**Published:** 2025-07-24

**Authors:** Man Gong, Lili Zhu, Bingdi Cui, Cong Ling, Xiaoqian Liu, Zhimin Wang, Liping Dai

**Affiliations:** ^1^ Henan Collaborative Innovation Center of Research and Development on the Whole Industry Chain of Yu-Yao, Henan University of Chinese Medicine, Zhengzhou, Henan, China; ^2^ Academy of Chinese Medical Sciences, Henan University of Chinese Medicine, Zhengzhou, Henan, China; ^3^ Institute of Chinese Materia Medica, China Academy of Chinese Medical Sciences, Engineering Technology Research Center for Comprehensive Development and Utilization of Authentic Medicinal Materials from Henan, Zhengzhou, Henan, China; ^4^ Institute of Chinese Materia Medica, China Academy of Chinese Medical Sciences, Beijing, China

**Keywords:** atherosclerosis, *Eucommia ulmoides* leaves, EUL 50, inflammation, NLRP3, autophagy

## Abstract

**Background:**

Atherosclerosis (AS) is a prevalent systemic disease, with its morbidity and mortality rates escalating globally. Traditional Chinese medicine (TCM), characterized by its multi-pathway and multi-target approach, offers distinct advantages in the diagnosis and treatment of atherosclerosis. *Eucommia ulmoides* (*E. ulmoides*) leaves, known for their medicinal and nutritional benefits, exhibit multi-targeted mechanisms against AS, although their precise pharmacological basis and molecular mechanisms are not fully understood. In this study, two active metabolites were isolated from the leaves of *E. ulmoides* leaves. Preliminary pharmacodynamic evaluation demonstrated that one metabolite, referred to as *Eucommia ulmoides* leaves (EUL 50), exhibited superior efficacy. This research focuses on the effects of EUL 50 on AS.

**Methods:**

The study assessed the impact of EUL 50 supplementation on AS in male Wistar rats, which were administered EUL 50 at doses of 70 mg/kg (low) and 140 mg/kg (high) to evaluate effects on lipid metabolism, NOD-, LRR- and pyrin domain-containing protein 3 (NLRP3) inflammasome activity, and autophagy. Additionally, the effect of EUL 50 on NLRP3 inflammasomes and autophagy was examined in an oxidized low-density lipoprotein (ox-LDL)-induced THP-1 foam cell model.

**Results:**

EUL 50 significantly reduced serum inflammation markers, including tumor necrosis factor-α (TNF-α), interleukin-6 (IL-6), interleukin-1β (IL-1β), vascular cell adhesion molecule-1 (VCAM-1), intercellular cell adhesion molecule-1 (ICAM-1), and matrix metalloproteinase-9 (MMP-9). It also lowered the levels of triglycerides (TGs), total cholesterol (TC), and low-density lipoprotein cholesterol (LDL-C) in the blood while increasing high-density lipoprotein cholesterol (HDL-C) levels in AS rats. Histopathological analysis of liver tissue, along with liver Oil Red O staining and aortic hematoxylin and eosin (HE) results, indicated that EUL 50 improved lipid accumulation. Furthermore, EUL 50 inhibited ox-LDL-induced foam cell formation and cholesterol (TC) accumulation while also suppressing the levels of TNF-α, IL-6, and IL-1β. Additionally, EUL 50 inhibited the expressions of NLRP3, ASC, caspase-1, and p62 proteins in AS rats and foam cells, thereby hindering the progression of AS.

**Conclusion:**

EUL 50, an active metabolite from *E. ulmoides* leaves, demonstrates potential in preventing and treating AS through autophagy-mediated regulation of NLRP3 inflammasomes. These findings support the potential development of health products derived from *E. ulmoides* leaves.

## Graphical Abstract

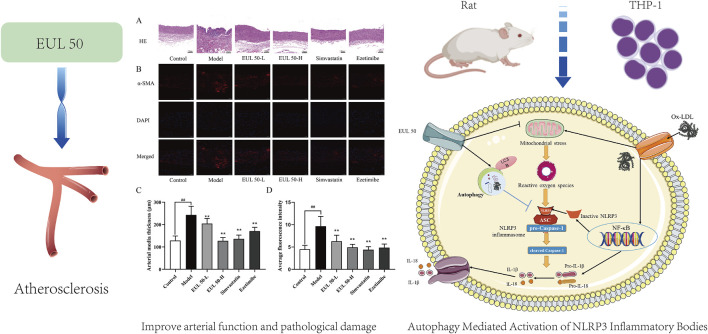

## 1 Introduction

Atherosclerosis (AS), a prevalent contributor to the pathogenesis of cardiovascular disease (CVD), primarily manifests in regions of disturbed laminar flow within the middle arteries and aorta, particularly at arterial bifurcations and branches (Tabas et al., 2015; Libby et al., 2019). AS is characterized by multifaceted pathogenesis, and current therapeutic drugs can only improve symptoms without curing this disease. AS requires long-term medication treatment, which causes a substantial amount of pain for patients and a heavy financial burden on their families and even society. It is important to search for drugs that can prevent AS and elucidate their mechanism of action to improve health and reduce the economic burden of AS on patients’ families and society.

Disordered lipid metabolism and inflammation are key factors that contribute to AS, and these factors interact with each other (Tuñón et al., 2019). Low-density lipoprotein cholesterol (LDL-C) crucially marks the progression of AS and is taken up by vascular endothelial cells, which are converted into foam cells, promoting plaque formation. Consequently, reducing LDL-C levels is imperative for the prevention and management of AS (Chistiakov et al., 2017). Inflammation accelerates the progression of AS and primarily drives plaque rupture. NOD-, LRR- and pyrin domain-containing protein 3 (NLRP3)-mediated activation of inflammatory vesicles increases the release of pro-inflammatory factors, which promote lipid deposition and foam cell accumulation, and NLRP3 is thus associated with the progression of AS (Duewell et al., 2010; He et al., 2012; Jin and Fu, 2019). Autophagy promotes smooth muscle cell survival and strengthens the fibrous cap, improving plaque stability (Osonoi et al., 2018). Autophagy serves as a significant route for the degradation and clearance of inflammatory vesicles. Phagocytic vesicles in the cytoplasm engulf NLRP3 oligomers to form autophagosomes, which fuse with lysosomes to form autophagic lysosomes to achieve inflammatory vesicle clearance. Autophagy serves as an endogenous inhibitory mechanism of action against NLRP3 inflammatory vesicles as autophagy activation inhibits the activation of these vesicles (Satish and Agrawal, 2020). Researchers discovered that autophagy modulated the activation of the NLRP3 inflammasome in a mouse model deficient in autophagy-related protein 5 (ATG5) (Shi et al., 2012).

*Eucommia ulmoides* (*E*. *ulmoides*) leaves are extensively utilized in traditional Chinese medicine (TCM) and the health food and functional food industries. *E. ulmoides* leaves are abundant and are considered a representative functional food in China and Japan (He et al., 2014; Zhu and Sun, 2018; Li et al., 2022). Long-term consumption of tea made from *E. ulmoides* leaves lowers blood pressure, blood lipid, and blood sugar levels; enhances immunity; and combats fatigue (Kobayashi et al., 2012; Ishimitsu et al., 2021; Huang et al., 2023). *E. ulmoides* leaves serve as a traditional medicine specific to diabetes in South Korea (Kim et al., 2004). In addition, *E. ulmoides* leaves can increase immunity in livestock and poultry, reduce antibiotic usage, improve meat and egg quality, increase piglet growth, and support intestinal function (Ding et al., 2020; Yang et al., 2024).

The therapeutic application of *E*. *ulmoides* leaves was first documented in “Tujing Ben Cao.” Classic records of TCM, such as “The Compendium of Materia Medica,” written during the Ming Dynasty, and “Recipes of Guangqun,” written during the Qing Dynasty, report that young *E. ulmoides* leaves are edible. *E. ulmoides* leaves have demonstrated unique benefits in treating metabolic diseases; for instance, they can regulate blood pressure (Hosoo et al., 2017), decrease blood lipid (Hao et al., 2016) and blood sugar levels (Jin et al., 2010), and prevent AS (Lee et al., 2018). Iridoids, flavonoids, and phenolic acids, which reduce atherosclerotic inflammation and lipid buildup, are abundant in *E. ulmoides* leaves. For example, quercetin mitigates atherosclerotic inflammation by restricting the activation of the NLRP3 inflammasome (Li et al., 2021). Chlorogenic acid can inhibit lipid accumulation (Duan et al., 2019; Zhang et al., 2020). Two metabolites EUL 50 and EUL 95, were identified from an aqueous extract of *E. ulmoides* leaves. EUL 50 more effectively reduced lipid accumulation than EUL 95 in a HepG2 cell model. Compositional analysis revealed that the main metabolites of EUL 50 are quercetin glycosides and caffeoylquinic acid (Gong et al., 2022). EUL 50 may decrease blood lipid levels by activating autophagy and triggering the expression of peroxisome proliferator-activated receptor γ (PPARγ) (Gong et al., 2022).

AS is a CVD characterized by long-term chronic inflammation. *E. ulmoides* leaf extract is capable of reducing the area of atherosclerotic plaque lesions (Hashikawa-Hobara et al., 2020), necessitating the classification of its metabolites and the investigation of the relevant mitigating mechanism of action against AS. The study adopted oxidized low-density lipoprotein (ox-LDL)-induced THP-1 foam cells to assess the efficacy of EUL 50 and EUL 95 in preventing AS, suggesting EUL 50 as the most effective active metabolite. We also established *in vivo* and *in vitro* models of AS for the assessment of the impact of EUL 50 on AS. Additionally, we investigated whether EUL 50 affects AS by modulating autophagy and NLRP3 inflammasome activation. The study results theoretically benefit the development of drugs derived from *E. ulmoides* leaves.

## 2 Materials and methods

### 2.1 Experimental samples

The study used *E. ulmoides* leaves sourced from Henan Golden Eucommia Agricultural Technology, which were confirmed as *E. ulmoides* Oliver by Professor Li-Ping Dai of Henan University of Chinese Medicine. EUL 50 and EUL 95 were first obtained from an extract of *E. ulmoides* leaves, and their chemical compositions were then analyzed (Gong et al., 2022).

### 2.2 Establishment of an AS rat model

Forty male Wistar rats weighing 190 ± 20 g were housed under controlled conditions: temperature, 22°C ± 1°C; relative humidity, 50%–60%; and a 12-h light–dark cycle. The rats were fed for 7 days to allow them to adjust to the environment. Body weight was then used to classify them into five groups: the control group (control, 0.5% carboxymethylcellulose), model group (0.5% carboxymethylcellulose), low-dose EUL 50 group (EUL 50-L, 70 mg/kg), high-dose EUL 50 group (EUL 50-H, 140 mg/kg), and simvastatin group (simvastatin, 5 mg/kg). Each group consisted of eight rats, and the rats in each group were numbered and weighed. Prophylactic administration was carried out concurrently with modeling for 12 weeks. A model of AS was generated as follows: vitamin D3 (VD3, Shanghai General Pharmaceutical, Shanghai, China) was injected into the rats at a dose of 600,000 IU/kg before modeling and 100,000 IU/kg in weeks 2, 4, 6, 8, and 10 after modeling. The animals were fed a high-fat diet (HFD, encompassing 0.2% propylthiouracil, 0.5% sodium cholate, 2.5% cholesterol, 10% lard, 10% egg yolk powder, and 76.8% normal chow) for 12 weeks. Two rats were randomly selected and euthanized at the end of the 11th week after modeling, and HE staining was used to assess successful model establishment. The rats were weighed once per week.

### 2.3 Specimen collection

One hour following the last administration, the rats were anesthetized, and blood was collected from the abdominal aorta; subsequently, the organs were dissected. Blood was incubated at room temperature to allow natural coagulation and centrifuged for 5 min (3,000 r/min), and the upper layer, consisting of serum, was collected and used to measure biochemical indices.

### 2.4 Determination of serum lipid levels

Commercially available kits (Nanjing Jiancheng Institute of Biological Engineering, Nanjing, China) were used to examine the serum levels of TC, TG, HDL-C, and LDL-C in the rats. The AS index (*AI*) was calculated as [TC-HDL] ÷ HDL (Deng et al., 2023), and rats with an *AI* <4 were considered normal.

### 2.5 Determination of serum inflammatory factor levels

The serum levels of inflammatory cytokines in rats, including tumor necrosis factor-α (TNF-α), vascular cell adhesion molecule-1 (VCAM-1), intercellular cell adhesion molecule-1 (ICAM-1), interleukin-6 (IL-6), interleukin-1β (IL-1β), and matrix metalloproteinase-9 (MMP-9), were determined using kits from Guangzhou Darwin Biotechnology, Guangzhou, China.

### 2.6 HE staining

The liver tissue and aorta removed from the rats were washed. After fixation in paraformaldehyde (PFA) and paraffin embedding, tissues were dehydrated in ethanol, cleared in xylene, and sectioned. The tissue slices were stained with an HE solution and sealed on slides. A light microscope was used for the observation of liver tissues’ pathological changes.

### 2.7 Oil Red O staining

The liver was removed from each rat and washed with cold saline. The livers were cut into 2 × 2 cm pieces to undergo 24 h of fixation in 4% PFA. Then, the liver tissue sections were rinsed under gently running water for 30 min to remove residual 4% PFA and subsequently incubated in a 60% isopropanol solution for 15 min. After approximately 3 h of Oil Red O staining, the liver tissue sections were soaked 5‒6 times with 60% isopropanol, then sealed, and observed under a light microscope.

### 2.8 Immunohistochemical analysis

Rat aortic tissues were removed, washed with cold saline, and dried using filter paper. Two-centimeter sections of the aorta were excised and fixed in 4% PFA for 24 h, followed by paraffin embedding and detection of p62 and NLRP3 protein expressions.

### 2.9 Immunofluorescence analysis

The aortas were embedded in paraffin, followed by three washes with PBS and 1 h of sealing using a 0.3% solution of bovine serum albumin at ambient temperature. The sections were incubated overnight with an α-smooth muscle actin (α-SMA) antibody at 4°C. The sections were then treated with a secondary antibody (anti-rat IgG) and stained with DAPI before being viewed and photographed under a fluorescence microscope.

### 2.10 Macrophage culture

THP-1 cells (Shanghai Institute of Cell Research, Chinese Academy of Sciences) were cultivated in DMEM supplemented with 10% FBS. Macrophages between passages 4 and 7 were used for subsequent experiments. The cells were inoculated in different plates and incubated overnight. THP-1 cells in the logarithmic growth phase, at a concentration of 4 × 10^5^/mL, were induced to differentiate into macrophages through 48 h of treatment with 100 μg/mL PMA (Beijing Solarbio Science & Technology, Beijing, China) in culture dishes or wells. Subsequently, the cells were treated using 50 μg/mL ox-LDL alone or in combination with varying concentrations of the experimental reagents.

### 2.11 Cytotoxicity of EUL 50 and EUL 95

Inoculated THP-1 cells in 96-well plates were stimulated with EUL 50 (1, 5, 10, 50, 100, or 200 μg/mL) or EUL 95 (1, 5, 10, 50, 100, or 200 μg/mL). The cells were incubated for 24 h and then incubated for an additional 2–4 h with the CCK-8 staining solution. Each sample’s absorbance was detected at 450 nm.

### 2.12 Foam cell lipid accumulation assay

Macrophages were cultured and treated with ox-LDL, then stained with Oil Red O and observed under an inverted microscope for imaging.

### 2.13 Determination of the TC content

TC levels in ox-LDL-treated foam cells were determined. EUL 50 (50, 100, and 200 μg/mL), EUL 95 (50, 100, and 200 μg/mL), and ox-LDL (50 μg/mL) were added, and the cells were co-cultured for 24 h. The cells and supernatants were collected for the assay. Intracellular TC levels were measured using a TC assay kit.

### 2.14 Inflammatory factor assay

To examine the impact of EUL 50 and EUL 95 on the secretion of inflammatory factors in foam cells, THP-1 cells were treated with EUL 50 (50, 100, and 200 μg/mL), EUL 95 (50, 100, and 200 μg/mL), the autophagy activator rapamycin, and the autophagy inhibitor chloroquine (CQ). Following a 24-h incubation period, the supernatant was collected after centrifugation at 2000 r/min, and the levels of IL-6, TNF-α, and IL-1β were determined.

### 2.15 Expression of proteins in the NLRP3 inflammatory vesicle and autophagy-associated proteins

Inflammatory vesicle pathways and autophagy-related proteins in rat arteries and foam cells were investigated through Western blotting. Cellular proteins (30 mg) were separated on a 10% SDS‒PAGE gel, transferred onto nitrocellulose (NC) membranes, and subsequently blocked with TBST buffer [10 mmol/L Tris (pH 7.4)] containing 5% BSA (with 150 mmol/L NaCl and 0.1% Tween-20 added at room temperature) for 2 h. After membrane transfer, the membranes were incubated with primary antibodies overnight at 4°C in a thermostatic shaker. Antibodies against NLRP3, IL-1β, p62, caspase-1, and GAPDH were used at a 1:1,000 dilution. ECL reagent was used for signal development, and protein bands were detected using a chemiluminescence image analysis system (Tanon 5200 Multi, Shanghai Tianneng Life Science Co., Ltd, Shanghai, China). Grayscale values were measured and quantified using ImageJ software.

### 2.16 Statistical analysis

Analyses of the experimental data were conducted using GraphPad Prism 8.0.1 software. Experimental data are presented as means ± standard deviations (x̅±SDs). One-way ANOVA was used to compare differences between groups. *P* < 0.05 was considered statistically significant.

## 3 Results

### 3.1 Screening of the optimal metabolite of *E. ulmoides* leaves for AS treatment

Cellular activity did not differ significantly between the EUL 50 or EUL 95 group and the control group when EUL 50 and EUL 95 were administered at concentrations ranging from 6.25 to 100 μg/mL. In addition, ox-LDL treatment increased intracellular TC accumulation in foam cells (*P* < 0.01), whereas EUL 50 (50, 100, or 200 μg/mL) or EUL 95 (100 or 200 μg/mL) treatment significantly decreased the TC content (*P* < 0.05 and *P* < 0.01). These findings indicated a notable increase in the release of TNF-α, IL-6, and IL-1β from ox-LDL-treated foam cells. Administration of EUL 50 (50, 100, or 200 μg/mL) significantly reduced the release of TNF-α, IL-6, and IL-1β (*P* < 0.01), whereas administration of EUL 95 at 50, 100, or 200 μg/mL significantly decreased the release of IL-6 (*P* < 0.01), and administration of EUL 95 at 100 or 200 μg/mL significantly increased TNF-α and IL-1β release (*P* < 0.05 and *P* < 0.01). EUL 50 treatment inhibited cholesterol accumulation and the release of inflammatory factors in foam cells more effectively than EUL 95 (Supplementary Figures S1–S5).

### 3.2 Effects of EUL 50 on the weight and lipid metabolism of AS rats

**FIGURE 1:**
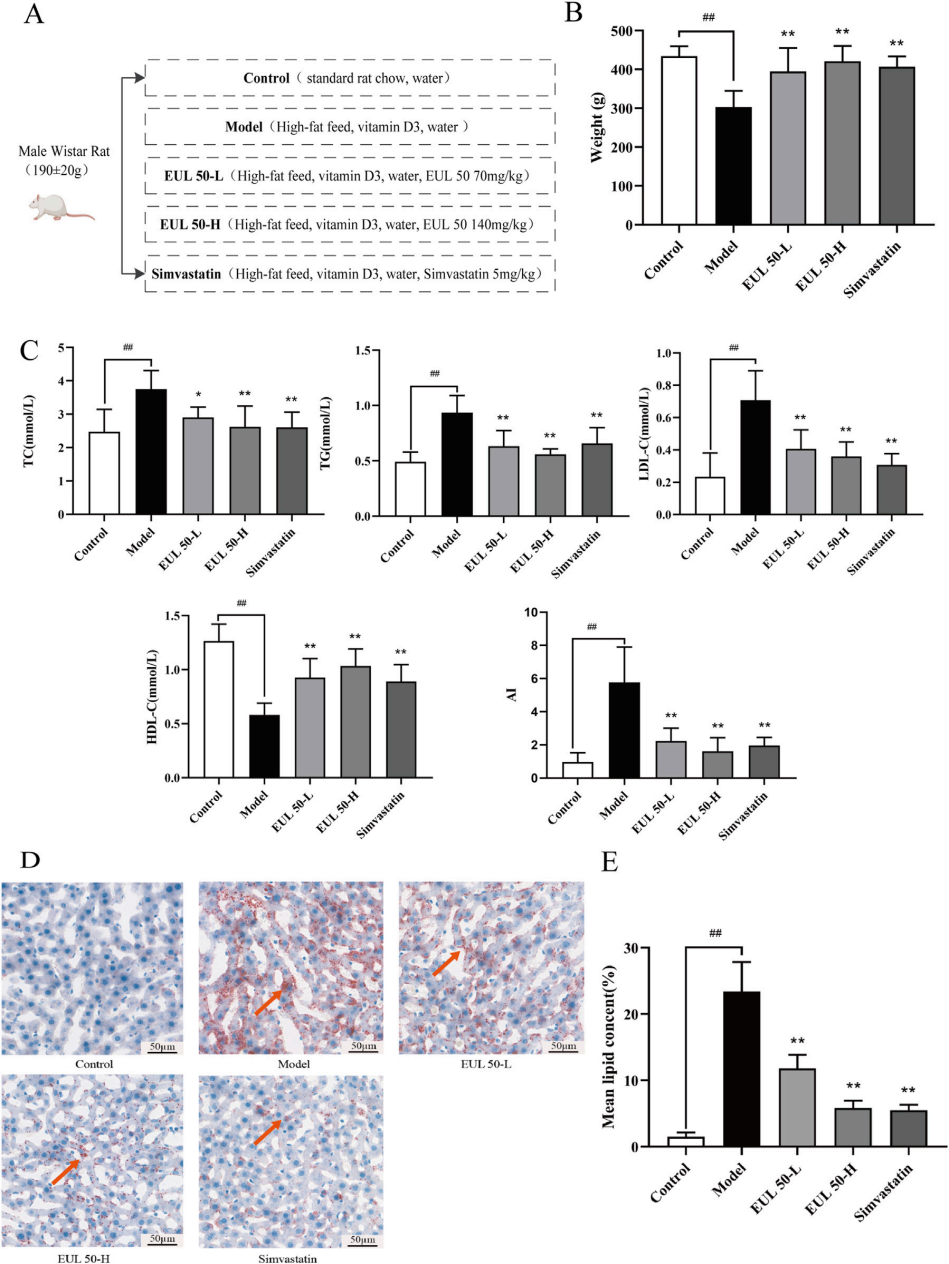
Effect of EUL 50 on weight and lipid metabolism in AS rats. **(A)** Schematic representation of the drug treatment protocol in each group. **(B)** Weights of the rats in each group. **(C)** Blood lipid levels and the AI in AS rats. **(D)** Liver Oil Red O staining. Red arrows indicate lipid droplets in the liver. **(E)** Statistical analysis of liver lipid accumulation. Note: Data are expressed as x̅ ± SD for each group, and n = 8. ^##^ *P* < 0.01 compared with the control group; ^*^ *P* < 0.05 and ^**^ *P* < 0.01 compared with the model group.

The diets of each group and the drugs that were administered are shown in a schematic representation in Figure 1A. The weights of the rats increased steadily with increasing feeding time, and the model group gained less weight than the control group. The control and experimental groups did not present significant differences in weight or hair density. The model group, fed an HFD and administered VD3, differed significantly from the control group; the model rats had yellow fur, exhibited signs of depression and decreased food intake, and had a lower weight (*P* < 0.01). The model group, which was both fed an HFD and administered VD3, had somewhat yellow fur, showed signs of depression, and exhibited significantly decreased food intake and reduced weight compared to the control group (*P* < 0.01), as indicated in Figure 1B. Additionally, the rats in the EUL 50-L, EUL 50-H, and simvastatin groups exhibited a remarkable weight loss compared to the model group at the statistical level (*P* < 0.01). The model group presented significantly elevated TC, TG, and LDL-C levels (*P* < 0.01) and significantly reduced HDL-C levels compared to the control group (*P* < 0.01). Furthermore, the EUL 50-L group exhibited remarkably lower serum TC (*P* < 0.05), TG, and LDL-C (*P* < 0.01) levels and considerably higher serum HDL-C level (*P* < 0.01) than those of the model group. Among blood lipid levels, the EUL 50-H group showed dramatically lower TC, TG, and LDL-C levels (*P* < 0.01) and remarkably higher HDL-C levels (*P* < 0.01). Similarly, the rats in the simvastatin group presented significantly reduced TC, LDL-C, and TG levels (*P* < 0.01) among blood lipids, and these changes were accompanied by significantly elevated HDL-C levels (*P* < 0.01). The model group presented significantly greater *AI* than that of the control group, and in the model group, an *AI* of 4 (*P* < 0.01), which is typical of a pathological state, was observed. The EUL 50-H, EUL 50-L, and simvastatin groups all presented dramatically lower *AI* than that of the model group, which was <4 (*P* < 0.01, Figure 1C). The model group exhibited more red-stained lipid droplets in the liver than the control group, as indicated in Figure 1D, indicating significantly greater lipid accumulation in the former group than the latter group (*P* < 0.01). Furthermore, the EUL 50-L, EUL 50-H, and simvastatin groups showed dramatically decreased lipid accumulation compared to the model group (*P* < 0.01), as indicated in Figure 1E.

### 3.3 Effect of EUL 50 on the levels of serum inflammatory factors in rats with AS

**FIGURE 2:**
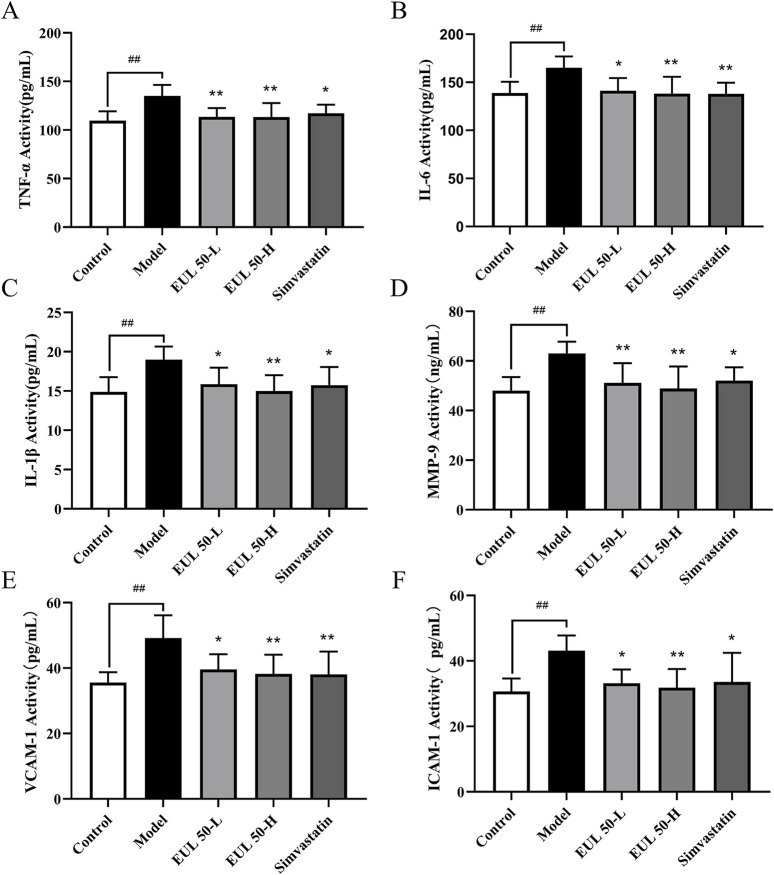
Effects of EUL 50 on the levels of inflammatory factors in AS rats. **(A)** TNF-α, **(B)** IL-6, **(C)** IL-1β, **(D)** MMP-9, **(E)** VCAM-1, and **(F)** ICAM-1. Note: Data are presented as x̅ ± SD, and n = 8. ^##^ *P* < 0.01 compared with the control group; ^*^ *P* < 0.05 and ^**^ *P* < 0.01 compared with the model group.

The model group presented significantly higher levels of inflammatory cytokines than those of the control group (*P* < 0.01). Furthermore, the EUL 50-L group showed remarkably lower TNF-α and MMP-9 levels than those the model group (*P* < 0.01), and the IL-1β, IL-6, ICAM-1, and VCAM-1 presented significantly lower levels in the EUL 50-L group (*P* < 0.01 and *P* < 0.05). The EUL 50-H group exhibited dramatically lower levels of inflammatory cytokines than those of the model group (*P* < 0.01). Furthermore, the simvastatin group presented dramatically lower IL-6 and VCAM-1 levels than those of the model group (*P* < 0.01), as well as reduced levels of TNF-α, IL-1β, MMP-9, and ICAM-1 (*P* < 0.05), as shown in Figure 2.

### 3.4 Effect of EUL 50 on pathological vascular changes in rats with AS

To investigate the impact of EUL 50 on pathological vascular changes, we conducted HE staining to observe changes in the tissue vasculature. Additionally, the protein expression level of α-SMA was investigated via immunofluorescence. Histological examination of the aorta using HE staining revealed that the control group presented a smooth and intact aorta intima, with well-organized smooth muscle cells (SMCs) in the media. Therefore, no obvious AS lesions were observed. The aortic intima of the model group appeared irregular, and substantial shedding of intimal cells was observed. The intima was broken in this group, together with the presence of elastic fibers and neointima and the production of intimal cells, foam cells, and fat vacuoles.

**FIGURE 3:**
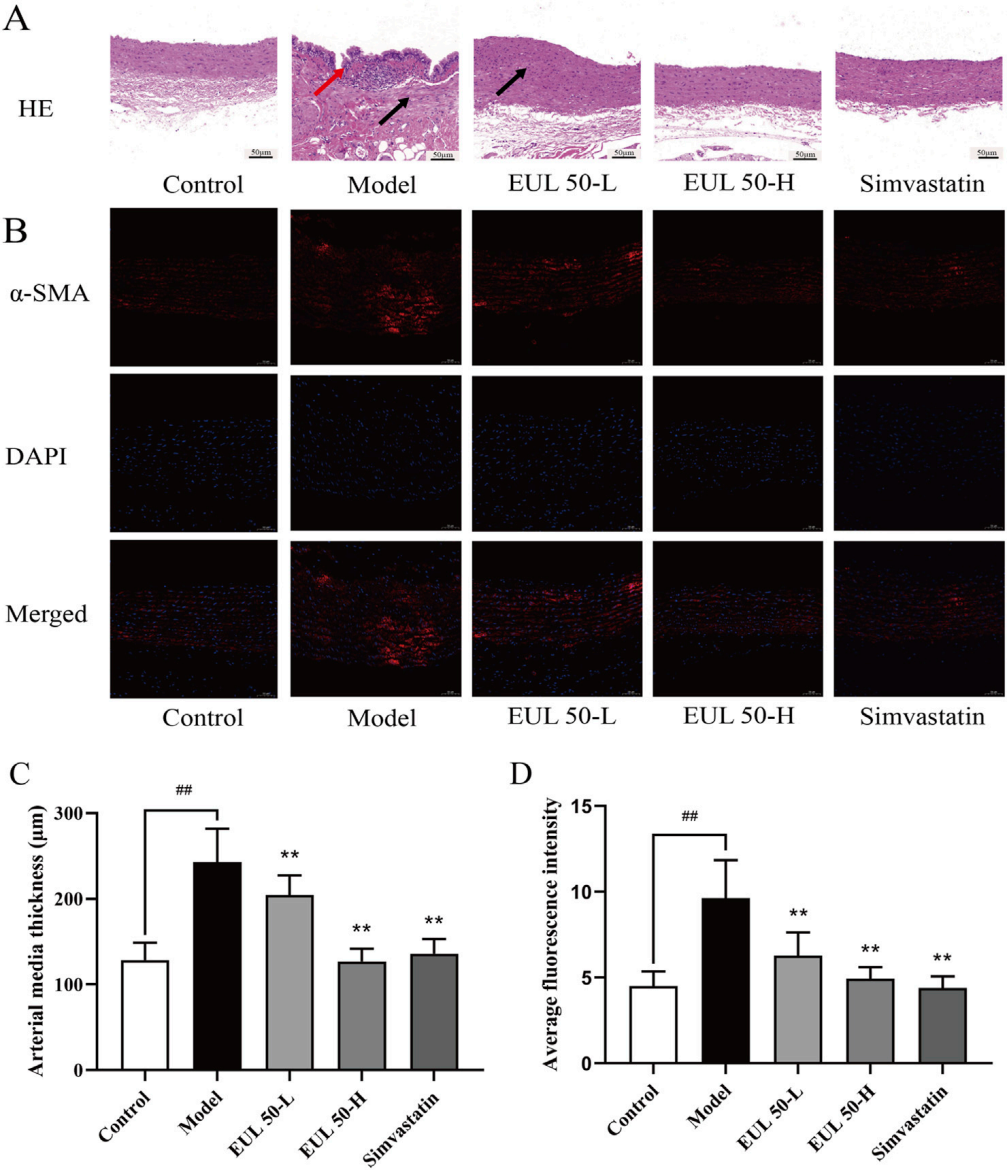
Protective effect of EUL 50 on the arteries of AS rats. **(A)** HE staining. Red arrows indicate irregular morphology of the aortic intima, and black arrows indicate that the SMCs in the media and adventitia had proliferated abnormally and were disordered. **(B)** Rat arterial α-SMA immunofluorescence staining. **(C)** Rat arterial medial thickness. **(D)** Rat arterial α-SMA fluorescence. Note: Data are expressed as x̅ ± SD, and n = 8. ^##^ *P* < 0.01 compared with the control group; ^**^ *P* < 0.01 compared with the model group.

Massive inflammatory cells had penetrated the tissue, accompanied by local shedding and rupture, and the SMCs in the media and adventitia had proliferated abnormally and were disordered. These changes, as shown in Figure 3A, reflect the typical pathology of AS. The aortic intima in rats in the EUL 50-L, EUL 50-H, and simvastatin groups was intact and showed no significant SMC proliferation, unlike that in the model group. Furthermore, the aortic intima of rats in the EUL 50-L, EUL 50-H, and simvastatin groups was intact, with no significant SMC proliferation in the media. The organization of the aortic intima was improved greatly, leading to significantly decreased thickness (*P* < 0.05), as shown in Figure 3C. According to immunofluorescence analysis (Figures 3B,D), the model group showed remarkably greater fluorescence intensity of α-SMA in the aorta than that of the control group (*P* < 0.01). In contrast, the EUL 50-L, EUL 50-H, and simvastatin groups had significantly lower fluorescence intensity of α-SMA than that of the model group (*P* < 0.01).

### 3.5 EUL 50 inhibits NLRP3 inflammasome activation in rats with AS

**FIGURE 4:**
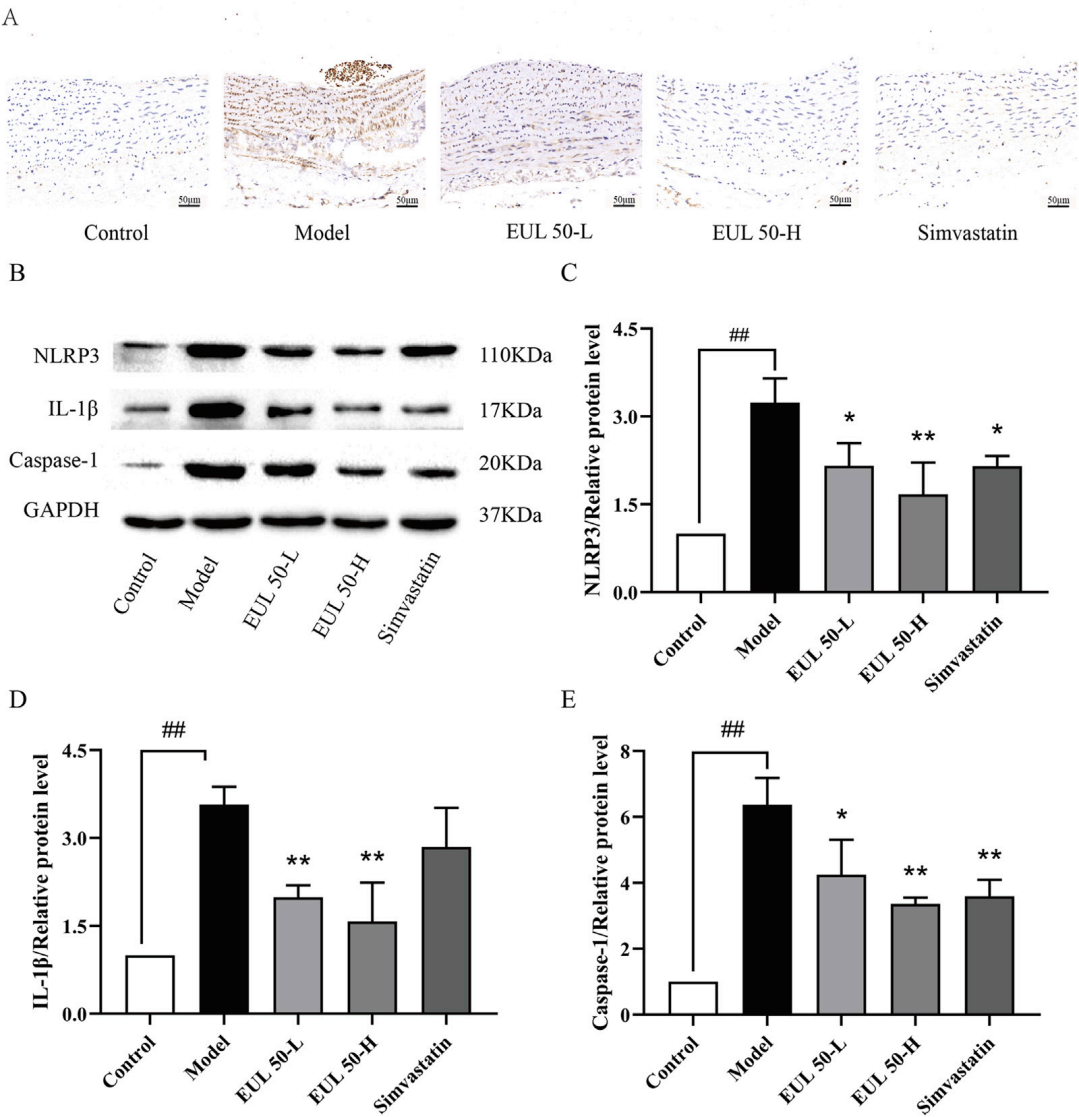
Effects of EUL 50 on the expression of NLRP3 inflammasome-related proteins in AS rats. **(A)** Rat arterial NLRP3 immunohistochemistry. **(B)** Rat arterial NLRP3-related protein bands. **(C)** Statistical analysis of rat arterial NLRP3 protein expression. **(D)** Statistical analysis of rat arterial IL-1β protein expression. **(E)** Statistical analysis of caspase-1 protein expression in rat arteries. Note: Data are expressed as x̅±SD, n = 3. ^##^ *P* < 0.01 compared with the control group; ^*^ *P* < 0.05 and ^**^ *P* < 0.01 compared with the model group.

We then assessed the impact of EUL 50 on protein expression in the NLRP3 inflammasome to verify its therapeutic mechanism of action in the rats with AS. Immunohistochemical analysis assisted in detecting the NLRP3 protein expression, as shown in Figure 4A. The model group presented higher NLRP3 levels in the arterial tissue than those of the control group, but the EUL 50-L, EUL 50-H, and simvastatin groups presented lower levels of NLRP3 in the arterial tissue than those of the model group. Western blotting revealed higher levels of NLRP3, IL-1β, and caspase-1 in the arterial tissues of the model group than those in the control group (*P* < 0.01). EUL 50-L and EUL 50-H treatment also significantly decreased protein expression compared to that in the model group (*P* < 0.05 and *P* < 0.01). NLRP3 and caspase-1 expression decreased in the simvastatin group compared to the model group (*P* < 0.05 and *P* < 0.01, Figures 4B–E). EUL 50 decreased the NLRP3 expression, suggesting the involvement of NLRP3 in AS.

### 3.6 EUL 50 promotes arterial endothelial autophagy in rats with AS

**FIGURE 5:**
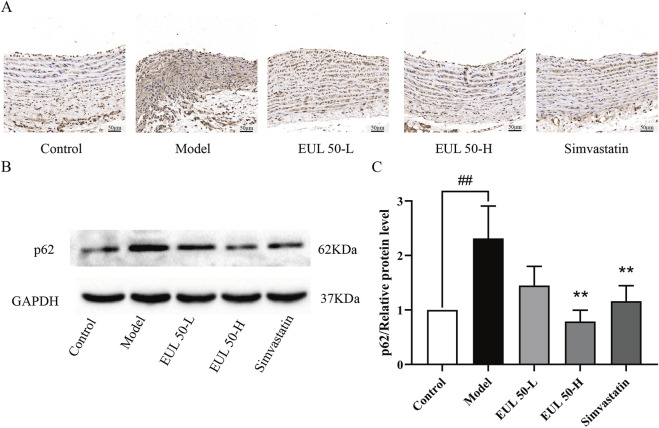
Effects of EUL 50 on the protein expression of p62/SQSTM1 in the aortas of AS rats. **(A)** Immunohistochemistry of rat arterial p62/SQSTM1. **(B)** Rat arterial p62/SQSTM1 protein band. **(C)** Statistical analysis of rat arterial p62/SQSTM1 protein expression. Note: Data are expressed as x̅ ± SD, n = 3. ^##^ *P* < 0.01 compared with the control group; ^**^ *P* < 0.01 compared with the model group.

We then assessed the impact of EUL 50 on p62/SQSTM1 expression to verify the mechanism of action by which EUL 50 protected the rats from AS. P62/SQSTM1 protein expression was detected through immunohistochemistry, as shown in Figure 5A. The model group presented a greater p62/SQSTM1 level than the control group. The EUL 50-L, EUL 50-H, and simvastatin groups presented lower p62/SQSTM1 levels in the arterial tissue than those of the model group. According to Western blotting to detect p62/SQSTM1 protein expression, the model group showed significantly decreased p62/SQSTM1 expression compared to the control group (*P* < 0.01), but EUL 50-H and simvastatin treatment decreased p62/SQSTM1 expression (*P* < 0.01, Figures 5B,C). The finding that EUL 50 decreased p62/SQSTM1 protein expression suggests the crucial role of autophagy in the effects of EUL 50 on AS in rats.

### 3.7 Effect of EUL 50 on ox-LDL-induced THP-1 foam cell formation and cholesterol accumulation

**FIGURE 6:**
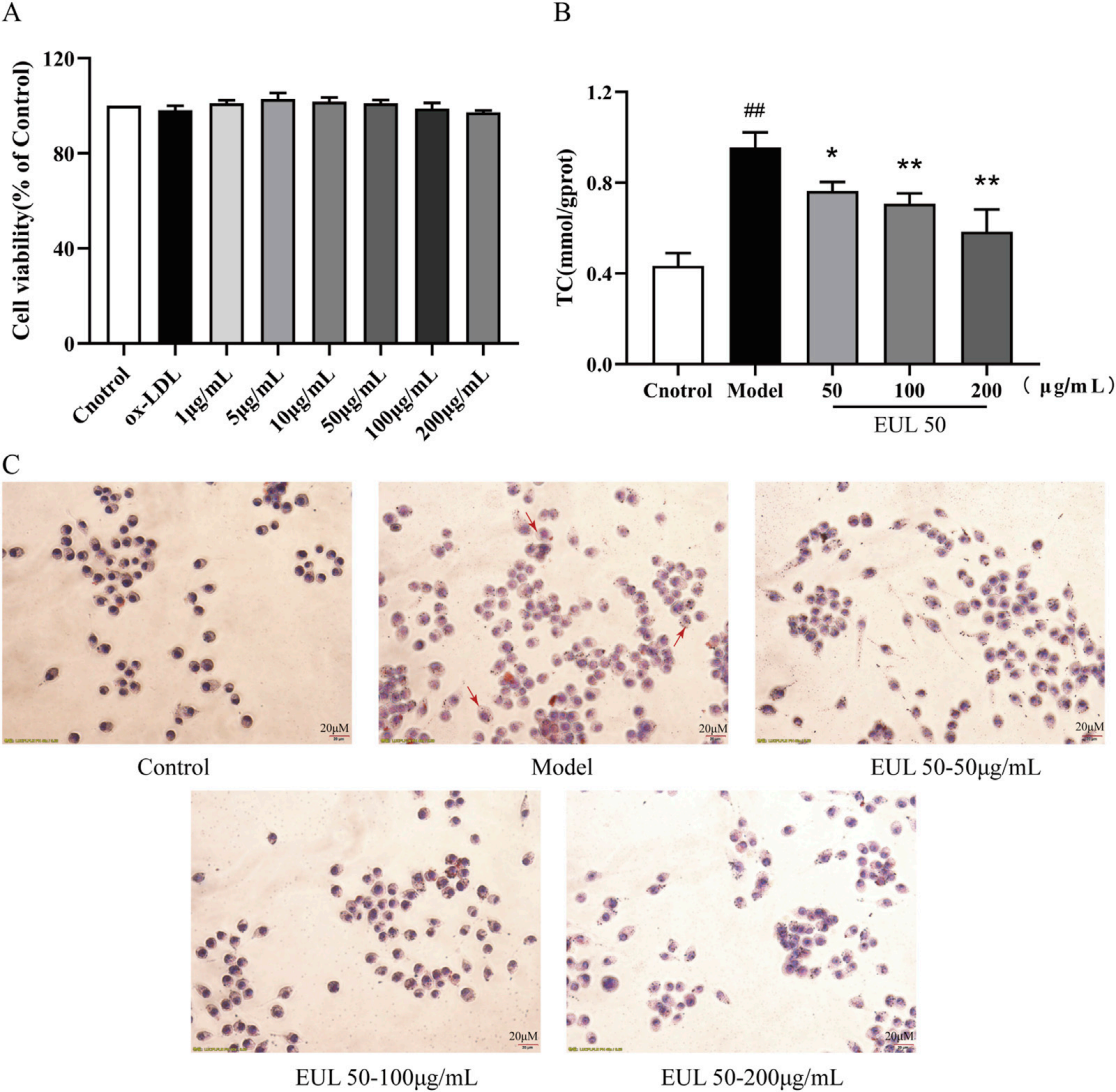
Effects of EUL 50 on ox-LDL-induced THP-1 foam cell formation and cholesterol accumulation. **(A)** Cytotoxic effects of EUL 50 and ox-LDL on THP-1 cells. **(B)** TC content in foam cells. **(C)** Oil Red O staining of foam cells (red arrows indicate intracellular lipid accumulation). Note: ^##^ *P* < 0.01 compared with the control group; ^*^ *P* < 0.05 and ^**^ *P* < 0.01 compared with the model group.

The effects of EUL 50 and ox-LDL on the viability of THP-1 cells were evaluated using a CCK-8 assay. When administered, EUL 50 (1–200 μg/mL) and ox-LDL (50 g/mL) did not significantly affect the viability, as shown in Figure 6A. However, ox-LDL treatment significantly increased intracellular TC accumulation (*P* < 0.01), as shown in Figure 6B. Furthermore, ox-LDL increased lipid deposition in macrophages, as indicated in Figure 6C, and many bright particles (indicated by red arrows) were observed in the model group. The treatment of foam cells with EUL 50 (50, 100, or 200 μg/mL) markedly diminished the intracellular TC levels (*P* < 0.05 and *P* < 0.01), indicating that EUL 50 significantly abrogated lipid accumulation triggered by ox-LDL.

### 3.8 Effect of EUL 50 on the release of inflammatory factors from foam cells via autophagy

**FIGURE 7:**
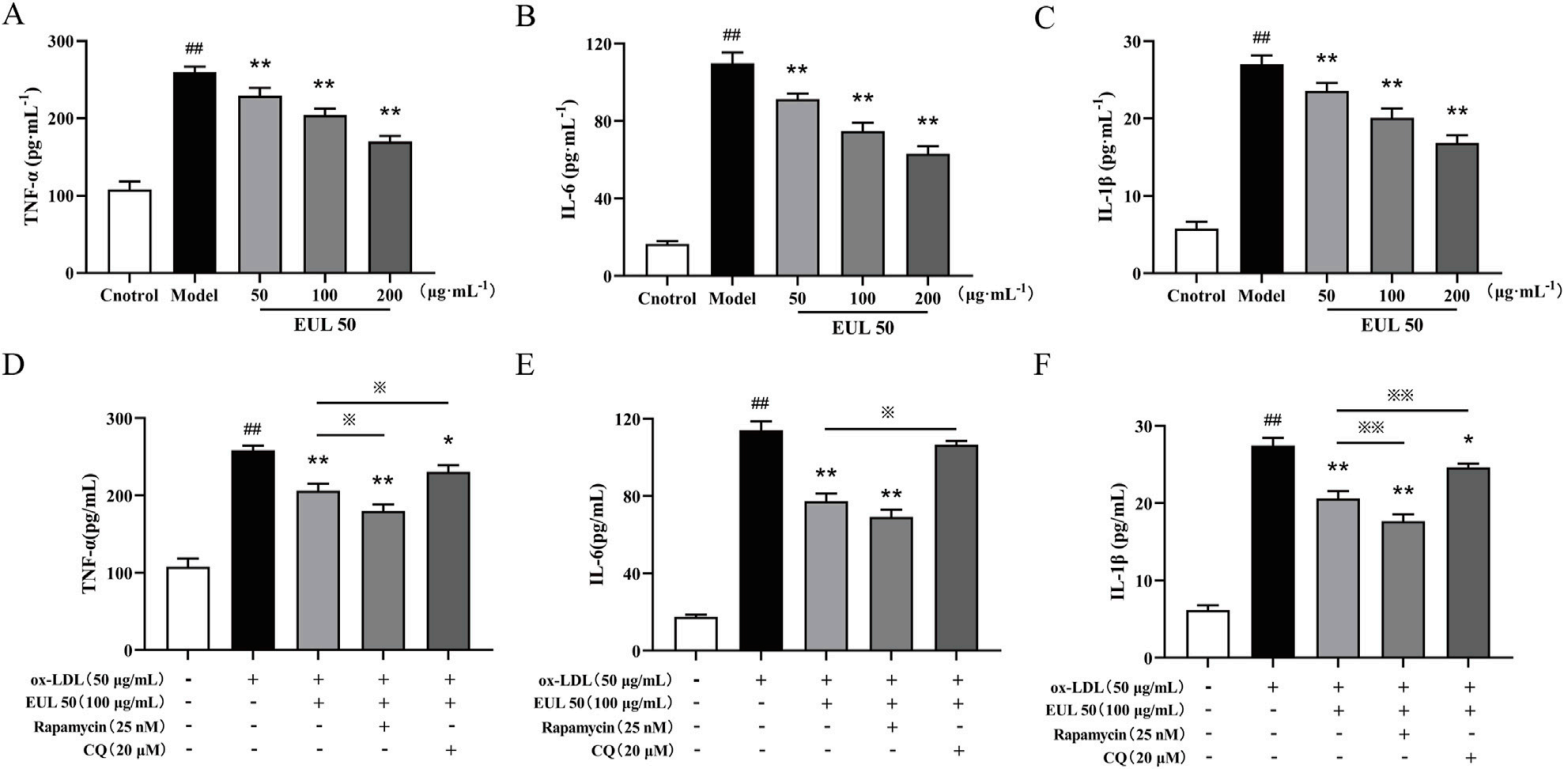
Effects of EUL 50 on the release of inflammatory factors from foam cells via autophagy. **(A)** Levels of released TNF-α. **(B)** Levels of released IL-6. **(C)** Levels of released IL-1β. **(D)** Levels of TNF-α released after autophagy activation and inhibition. **(E)** Levels of IL-6 released after autophagy activation and inhibition. **(F)** Levels of IL-1β released after autophagy activation and inhibition. Note: **(A)** TNF-α, **(B)** IL-6, and **(C)** IL-1β; compared with the control group, ^##^ *P* < 0.01; compared with the model group, ^*^ *P* < 0.05 and ^**^ *P* < 0.01; compared with the EUL 50 (100 μg/mL) group, ^※^ *P* < 0.05 and ^※※^ *P* < 0.01.

EUL 50 (50, 100, or 200 μg/mL) profoundly suppressed the secretion of TNF-α, IL-6, and IL-1β from foam cells stimulated by ox-LDL (*P* < 0.01), as shown in Figures 7A–C. Treatment with EUL 50 (100 μg/mL) and rapamycin (25 nM) significantly abrogated the release of TNF-α, IL-6, and IL-1β (*P* < 0.01); EUL 50 (100 μg/mL) and CQ (20 μM) treatment profoundly suppressed the release of TNF-α and IL-1β (*P* < 0.05). Additionally, compared with EUL 50 (100 μg/mL) treatment, EUL 50 (100 μg/mL) and rapamycin (25 nM) treatment profoundly suppressed the levels of TNF-α and IL-1β (*P* < 0.05 and *P* < 0.01), whereas EUL 50 (100 μg/mL) and CQ (20 μM) treatment profoundly suppressed the levels of TNF-α, IL-6, and IL-1β (*P* < 0.05 and *P* < 0.01), as shown in Figures 7D–F. The results elucidated that EUL 50 significantly attenuated the secretion of pro-inflammatory mediators in foam cells stimulated by ox-LDL, an effect intricately associated with autophagy. The autophagy activator rapamycin enhanced the anti-inflammatory effect of EUL 50, whereas the autophagy inhibitor CQ suppressed this effect.

### 3.9 EUL 50 inhibits NLRP3 inflammasome activation by activating autophagy

**FIGURE 8:**
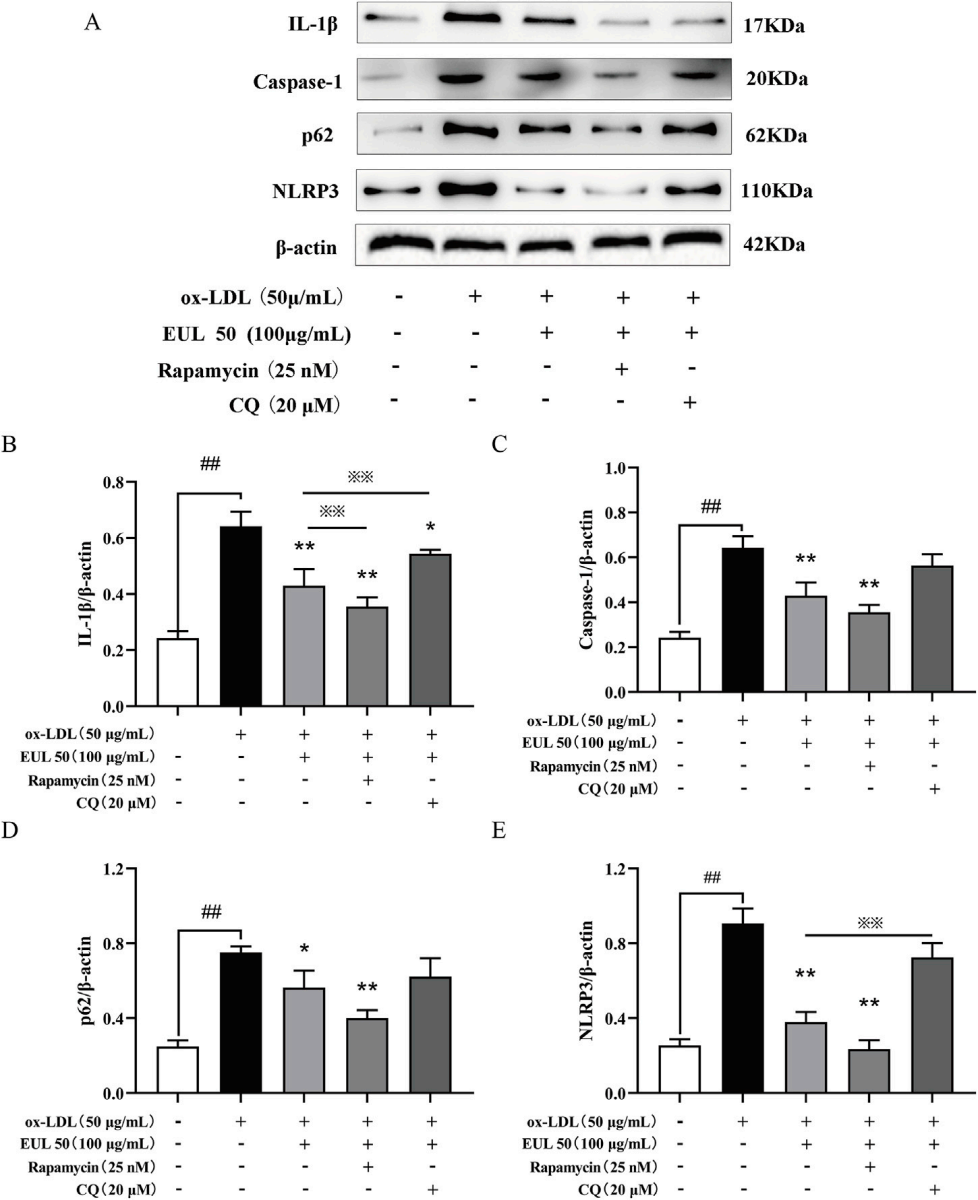
EUL 50 inhibits the expression of NLRP3 inflammasome proteins via autophagy. Note: **(A)** NLRP3 inflammasome-related protein bands. Statistical analysis of **(B)** IL-1β grayscale values, **(C)** caspase-1 grayscale values, **(D)** p62/SQSTM1 grayscale values, and **(E)** NLRP3 grayscale values. Compared with the control group, ^##^P < 0.01; compared with the model group, ^*^ *P* < 0.05 and ^**^ *P* < 0.01; compared with the EUL 50 (100 μg/mL) group, ^※^ *P* < 0.05 and ^※※^ *P* < 0.01.

To elucidate the correlation between the inhibitory effects of EUL 50 on autophagy and the activation of the NLRP3 inflammasome, we used rapamycin (25 nM) and chloroquine (CQ, 20 μM) as modulators. The protein levels of NLRP3, caspase-1, IL-1β, and p62/SQSTM1 were significantly decreased by the combined treatment with rapamycin (25 nM) and EUL 50 (100 μg/mL) compared to those after treatment with ox-LDL alone (*P* < 0.01). This finding suggests that the anti-inflammatory effects of EUL 50 were enhanced by co-treatment with rapamycin. Conversely, co-treatment with CQ (20 μM) and EUL 50 (100 μg/mL) led to the upregulation of NLRP3, caspase-1, IL-1β, and p62/SQSTM1 expressions. Notably, the level of IL-1β was markedly diminished in the co-treated group compared to the EUL 50 (100 μg/mL) group (*P* < 0.01), as shown in Figure 8. These findings indicated that EUL 50 (100 μg/mL) elicits an anti-inflammatory response through autophagy-mediated modulation of NLRP3 inflammasome activation.

## 4 Discussions

*E. ulmoides* leaves, which play an important role in TCM, have been used both as food and medicine. Because of their unique medicinal efficacy and nutritional value, *E. ulmoides* leaves are not only widely used in TCM but also have become a popular raw material in the health food industry, a dietary supplement, and a healthcare product. Therefore, the medicinal effects of *E. ulmoides* leaves should be investigated in greater depth to guarantee the improvement of the added value of *E. ulmoides* leaf-derived products, and the commercial use of *E. ulmoides* should be expanded to further increase the economic value of this product. The search for effective drugs for AS with few long-term side effects has become a top priority in the study. *E. ulmoides* leaf extract reduced body weight, blood pressure, and aortic intima–media thickness in HFD-fed rats. According to our study, administering *E. ulmoides* leaf extract for a long time may inhibit the development of AS (Hosoo et al., 2017). An ethanol extract of *E. ulmoides* leaves inhibited the development of AS induced by HFD feeding and VD3 administration, increased the aortic wall thickness, and reduced foam cell accumulation and smooth muscle hyperplasia (Hashikawa-Hobara et al., 2020). A flavonoid-rich diet may reduce the risk of CVD. Furthermore, research on quercetin glucoside derivatives, which improve lipid metabolism and have anti-inflammatory effects, has increased.

Quercetin-3-O-glucoside was reported to decrease plasma cholesterol and insulin levels, decrease PCSK9 levels, and increase LDLR and PCSK9 expressions in the liver and pancreas (Mbikay et al., 2018). Eicosapentaenoic acid esters of quercetin-3-O-glucoside were used to effectively treat hyperlipidemia by reducing inflammation and decreasing lipid levels (Sekhon-Loodu et al., 2015). In recent pharmacological studies, chlorogenic acid or its metabolites inhibited ox-LDL-induced lipid accumulation in the serum of ApoE^−/−^ mice and decreased the occurrence of AS, and these effects were primarily related to the powerful antioxidant properties of chlorogenic acid. These antioxidant effects resulted from lipid peroxidation inhibition and free radical elimination, and chlorogenic acid also improved endothelial cell function by reducing free-radical formation from NO (Jung et al., 2017).

UPLC-PDA-ESI-MS analysis identified chlorogenic acid, cryptochlorogenic acid, neochlorogenic acid, quercetin-3-O-sambubioside, rutin, and isoquercitrin as the primary metabolites of EUL 50 from *E. ulmoides* leaves, which are primarily flavonoids and phenolic acids. Consistent with the analysis of the individual metabolites EUL 50 showed an effect against AS. Specifically, EUL 50 alleviated aortic lesions and reduced serum levels of inflammatory factors and lipids. AS is characterized by chronic inflammation, and areas of laminar flow disturbance in the medium and large arteries, especially arterial branch points and branches, are susceptible to AS (Tabas et al., 2015). AS is caused by the subendothelial accumulation of ox-LDL, which is produced following injury and causes endothelial cell activation (Kianmehr et al., 2022) and the subsequent recruitment of blood-derived monocytes to the lesion. Macrophages phagocytose ox-LDL, leading to the formation of foam cells that accumulate under the arterial intima, forming lipid streaks (Taleb, 2016; Malekmohammad et al., 2021), which is a hallmark of AS lesions. Macrophages trigger inflammation when necessary and suppress the inflammatory response when it is no longer needed (Dolfi et al., 2021). In AS, macrophages are predominantly polarized to an inflammatory phenotype (Swirski et al., 2016); this inflammatory phenotype promotes the development of vascular inflammation and AS (Taleb, 2016). Inflammation crucially affects AS and participates in all phases of this disease. The present study has shown that EUL 50 effectively reduced ox-LDL-triggered foam cell formation and intracellular cholesterol accumulation and effectively blocked the inflammatory response by ox-LDL-induced foam cells. EUL 50 significantly reduced lipid metabolism in the AS rats, and lowering LDL-C, an important risk factor for AS, reduces CVD events independently of other risk factors (Baigent et al., 2010). In the early stages of lesion initiation, LDL-C particles accumulate in the endothelium and are taken up by cells via the LDLR on the macrophage membranes to form ox-LDL. Serum cholesterol levels and the development of AS are closely associated, and ox-LDL levels are particularly strongly associated with AS (Miller et al., 2011). Ox-LDL elevated TNF-α, IL-1β, IL-6, ICAM-1, and VCAM-1 levels; as a result, HUVECs were subjected to vascular endothelial injury (Wang et al., 2018; Bian et al., 2020). EUL 50 significantly reduced LDL-C levels and decreased intra-arterial lipid deposition in AS rats, which supports its potential to improve vascular endothelial function in AS. Moreover, EUL 50 led to lower expression of inflammatory factors in AS rats, and the expressions of ICAM-1 and VCAM-1, possibly participating in plaque production and pathological changes in the early AS, are closely correlated with AS severity (Fotis et al., 2012). Under normal physiological conditions, ICAM-1 is absent or expressed at very low levels, primarily in endothelial cells and monocytes. However, upon stimulation with inflammatory factors, such as TNF-α, ICAM-1 is distributed in endothelial cells, and ICAM-1 gene transcription is promoted. This rapid increase in ICAM-1 expression increases monocyte adhesion to endothelial cells, thereby promoting inflammation and triggering the onset and progression of AS (Cai et al., 2020). Elevated MMP-9 levels are a predictive factor for CVD, likely due to their effects on AS, and are strongly associated with susceptibility to intraplaque hemorrhage in AS (Silvello et al., 2014). MMP-9 over-expression in AS plaques causes plaque instability by degrading the fibrous cap (Ketelhuth and Bäck, 2011). IL-6 polymorphisms can elevate the risk of AS, and IL-6 may mediate pro-inflammatory AS in patients suffering from chronic kidney disease (CKD) (Hassan et al., 2020). Our study revealed the EUL 50 serum levels of the inflammatory factors in rats with AS.

Autophagy regulates the activation of inflammatory vesicles, including NLRP3 inflammatory vesicles, via various mechanisms to maintain homeostasis *in vivo* (Saitoh and Akira, 2016). The excessive activation of inflammatory vesicles is prevented via the p62-dependent degradation of inflammatory vesicle metabolites. When NLRP3 inflammatory vesicles in monocytes are stimulated, ASC is recognized by p62, and NLRP3 inflammatory vesicle metabolites, including NLRP3 and ASC, co-localize with autophagosomes; this co-localization indicates that NLRP3 inflammasomes may be phagocytosed and degraded by autophagosomes (Shi et al., 2012). Treatment with autophagy activators significantly inhibited NLRP3 inflammatory vesicle activity and substantially decreased IL-1β secretion (Evans et al., 2018). Autophagy is closely associated with macrophage-derived foam cells and AS plaques; for instance, rockweed polysaccharides attenuated NLRP3 inflammatory vesicle activation in AS by increasing p62/SQSTM1-mediated autophagy (Cheng et al., 2020). Similar to the above findings, EUL 50 may inhibit inflammatory vesicle activation through the activation of autophagy.

## 5 Conclusion

Owing to their remarkable medicinal value, stable market demand, and broad economic value, *E. ulmoides* leaves have become a valuable cash crop with promising development prospects. Quercetin glycosides and caffeoylquinic acid, as the active metabolites of *E. ulmoides* leaves, can modulate lipid metabolism and inflammation. EUL 50 reduced serum inflammatory factor levels, blood lipid levels, and disordered hepatic lipid metabolism in AS rats, and immunohistochemistry revealed that EUL 50 administration resulted in a significant reduction in NLRP3, p62, and α-SMA expressions. Furthermore, EUL 50 inhibited NLRP3/ASC/caspase-1 inflammatory vesicle activation, activated autophagy to inhibit AS-related inflammatory responses, and improved vascular endothelial cell function to protect against AS. EUL 50 reduced intracellular cholesterol accumulation in foam cells and significantly decreased inflammatory factor release; EUL 50 may ameliorate AS by inhibiting inflammatory vesicle activation through the activation of autophagy. Therefore, EUL 50 may act as a dietary supplement for AS prevention and treatment. Our findings will contribute to the development of health products using *E. ulmoides* leaves theoretically.

## Data Availability

The original contributions presented in the study are included in the article/Supplementary Material; further inquiries can be directed to the corresponding authors.
