# Supplementary material for: The active metabolites of Eucommia ulmoides leaves alleviate atherosclerosis induced by a high-fat diet and VD3 in rats
Source: Front Pharmacol. 2025 Jul 24;16:1625200. doi: 10.3389/fphar.2025.1625200 (PMC12328328; doi:10.3389/fphar.2025.1625200)
Supplement: Supplementary file 1 [file DataSheet1.docx]

Supplementary Material

# Supplementary Figures and Tables

## Supplementary Figures


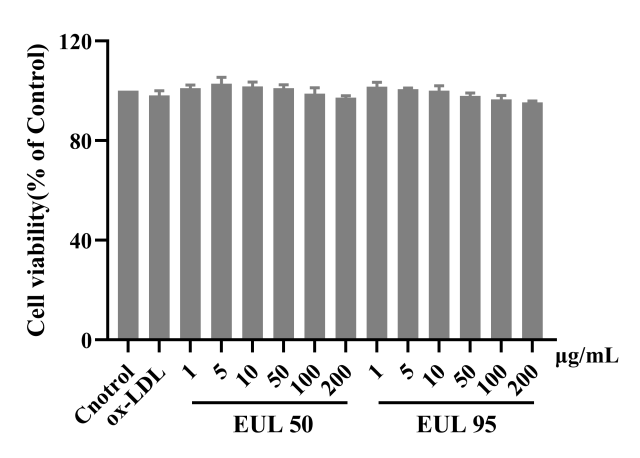


**Supplementary Figure 1** Cytotoxic effects of EUL 50, EUL 95 and ox-LDL on THP-1.


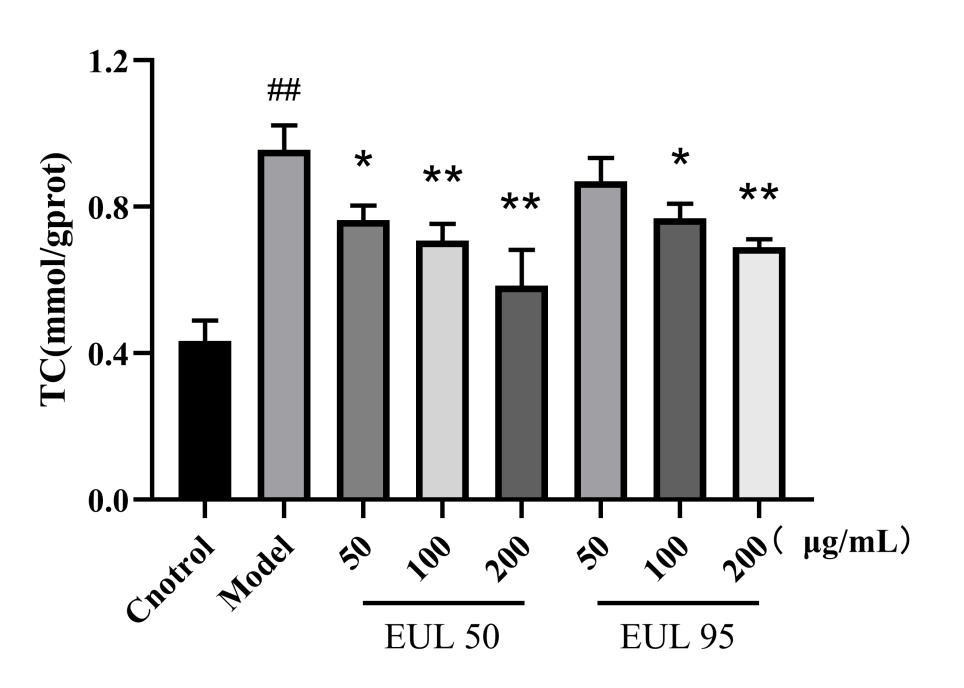


**Supplementary Figure 2** EUL 50 and EUL 95 inhibited ox-LDL-induced cholesterol accumulation in foam cells. Compared with Control group, ^##^*P*<0.01; compared with Model group, ^*^*P*<0.05, ^**^*P*<0.01.


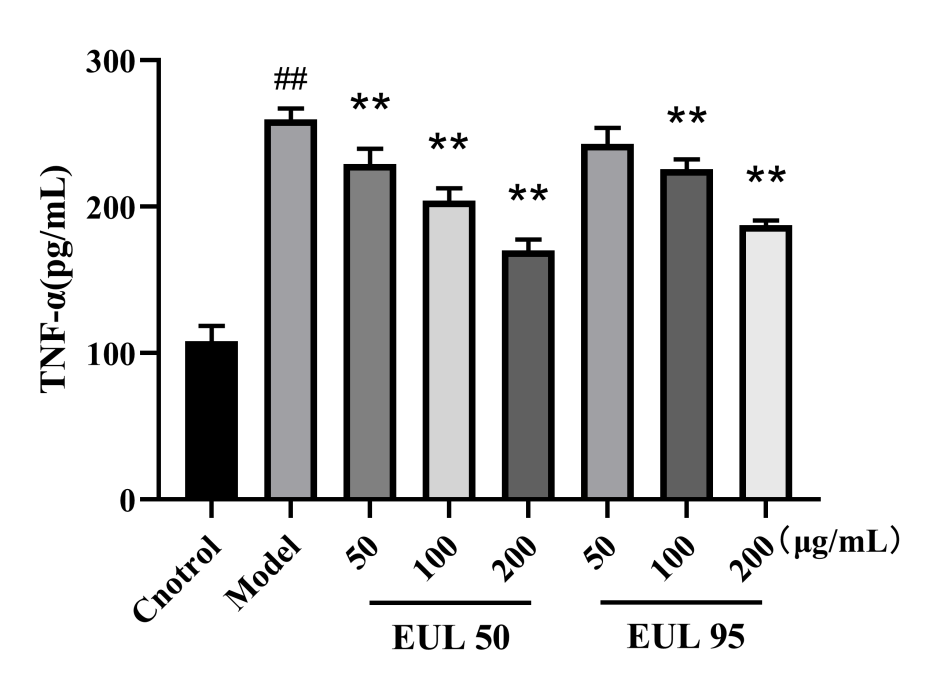


**Supplementary Figure 3** Effects of EUL 50 and EUL 95 on ox-LDL-induced release of inflammatory factor TNF-α from foam cells. Compared with Control group, ^##^*P*<0.01; compared with Model group, ^**^*P*<0.01; EUL 50 compared with the same dose of EUL 95, ^Δ^*P*<0.05 and ^ΔΔ^*P*<0.01.


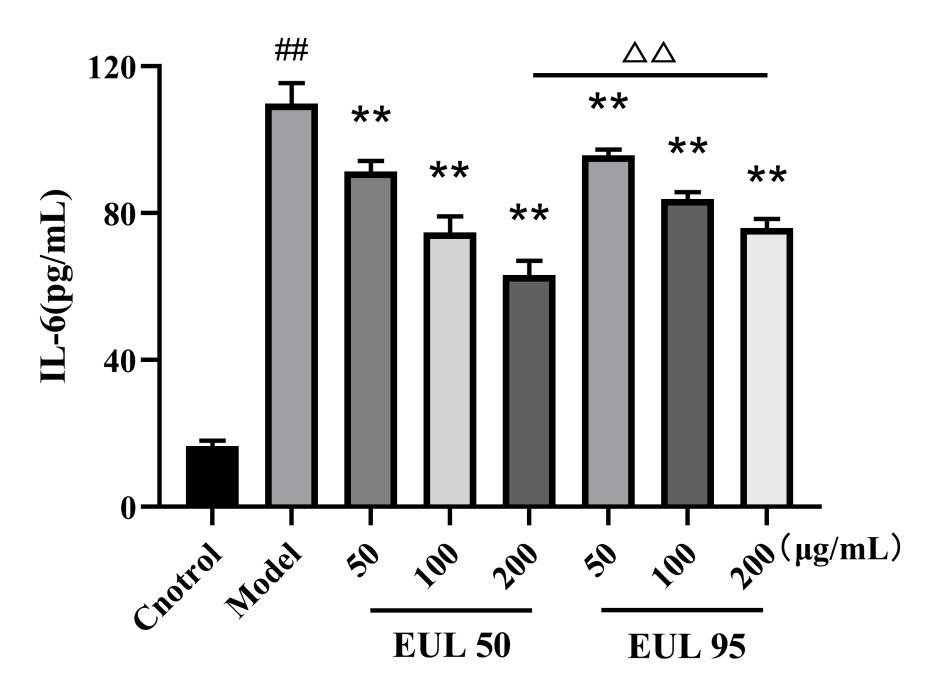


**Supplementary Figure 4** Effects of EUL 50 and EUL 95 on ox-LDL-induced release of inflammatory factor IL-6 from foam cells. Compared with Control group, ^##^*P*<0.01; compared with Model group, ^**^*P*<0.01; EUL 50 compared with the same dose of EUL 95, ^Δ^*P*<0.05 and ^ΔΔ^*P*<0.01.


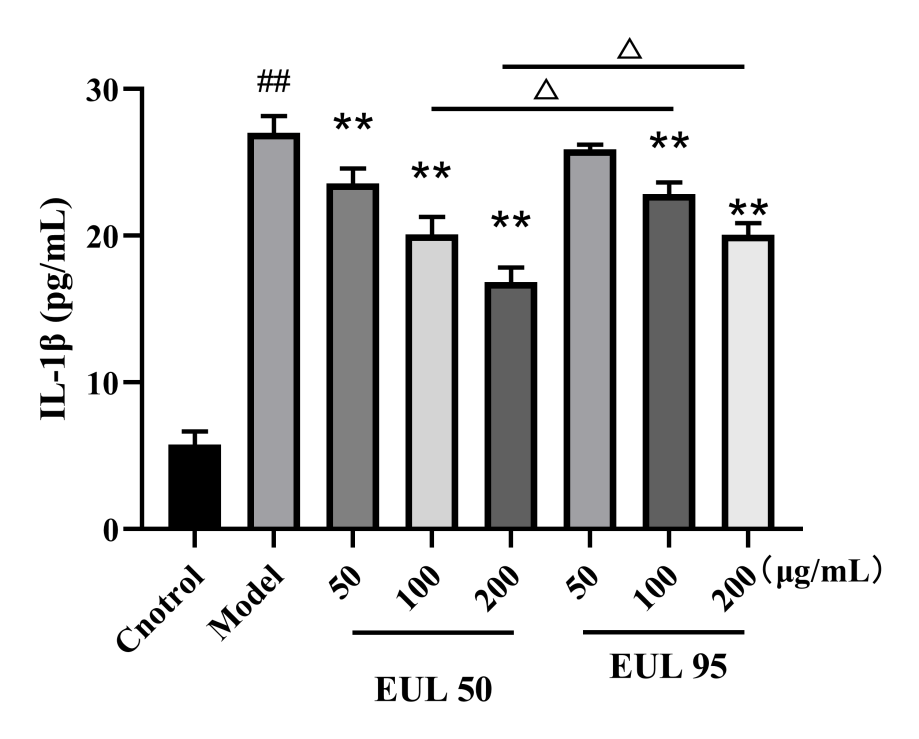


**Supplementary Figure 5** Effects of EUL 50 and EUL 95 on ox-LDL-induced release of inflammatory factor IL-1β from foam cells. Compared with Control group, ^##^*P*<0.01; compared with Model group, ^**^*P*<0.01; EUL 50 compared with the same dose of EUL 95, ^Δ^*P*<0.05 and ^ΔΔ^*P*<0.01.
